# Evaluation of the Cardiac Conduction System in Fibromyalgia Patients With Complaints of Palpitations

**DOI:** 10.7759/cureus.28784

**Published:** 2022-09-05

**Authors:** Serhat Günlü, Adem Aktan

**Affiliations:** 1 Cardiology, Dağkapı State Hospital, Diyarbakır, TUR; 2 Cardiology, Mardin Training and Research Hospital, Mardin, TUR

**Keywords:** holter-ecg, qt dispersion, p-wave dispersion, fibromyalgia, palpitations

## Abstract

Objective

This study aimed to investigate the influence of fibromyalgia syndrome (FMS) on the cardiac conduction system and assess patients’ palpitation complaints using 24-h ambulatory ECG (Holter {Maynard, MA: Northeast Monitoring, Inc.}) monitoring.

Methods

Ninety patients with FMS and 70 healthy controls were included in this research. ECG was performed on all participants, and ECG parameters were calculated. Holter monitoring was conducted, and the recordings were analyzed. The results of time-domain heart rate variability (HRV) were evaluated.

Results

The patient group’s mean age was 38.3±6.3 years. There were no statistically significant differences in demographic or laboratory parameters across the groups (p>0.05). The findings of a 24-h Holter ECG recording did not vary significantly between the groups (p=0.182). In the study group, the values for the standard deviation of R-R intervals (SDNN), low frequency (LF), and low/high frequency (LF/HF), which are known as HRV indicators, were substantially different between the two groups (p<0.05).

Conclusions

The study indicated that the risk of arrhythmias did not increase even though FM patients complained of palpitations.

## Introduction

Non-inflammatory soft-tissue rheumatism known as fibromyalgia syndrome is characterized by tiredness, prevalent chronic musculoskeletal soreness, morning stiffness, disturbed sleep, anxiety, and depression [1,2].

It can affect anyone at any age but is most prevalent in women aged between 20 and 45 years [3]. The diagnosis is delayed because multiple clinicians are involved in the complaints [4]. Due to clinicians' inability to fully interpret fibromyalgia syndrome (FMS), the average period required for a patient to receive a diagnosis after consulting a medical facility is two to three years [5]. During this period, many visits to the doctor have been made.

Patients report palpitations, chest pain, and back pain at the outpatient cardiology clinic. Patients with FMS who do not have heart pathology are often referred to a physiotherapy and rehabilitation specialist; however, patients with persistent palpitations are re-evaluated by a cardiologist [6]. ECG parameters are non-invasive predictors of arrhythmia risk [7]. Holter monitoring is frequently used as a non-invasive method for detecting arrhythmias that are not detected by ECG. Holter monitoring can also analyze heart rate variability (HRV) by measuring fluctuations over brief periods. Indicators of HRV may have a role in evaluating the relationship between brucellosis and dysfunctions of the autonomic nervous system.

In this study, we aimed to analyze the influence of FMS on the cardiac conduction system utilizing ECG parameters and to assess the efficacy of ECG parameters in predicting arrhythmias using Holter recording.

## Materials and methods

Study design and subjects

This cross-sectional study was conducted at Dagkapı State Hospital. The research sample consisted of patients who received a diagnosis between 2017 and 2021 by the American College of Rheumatology's guidelines (ACR, 2020). Included in the study were 98 patients who were diagnosed with FMS. Eight patients whose data were inaccessible were excluded from the research. Seventy healthy individuals were enrolled in the control group. FMS patients and healthy controls were examined in the cardiology department.

Study protocol

Using an electrocardiograph (model ECG-1350K; Tokyo, Japan: Nihon-Kohden Corporation), a 12-lead ECG was acquired at a rate of 25 mm/s and 10 mm/mV amplitude. To digitize the existing ECGs, a scanner was employed. Two cardiologists who were double-blinded to the study calculated and analyzed the T-peak to T-end (TpTe) time under 400x magnification. Significant and nearly complete agreement was observed between the two cardiologists' analyses (κ=0.861). In case of disagreement, a third cardiologist, who was double-blind to the study, was consulted. Using the Bazett formula, the QT interval and T-peak to T-end (TpTe) time for heart rate were corrected. These data were collected directly, as the QT interval, QTc interval, QRS, and T axis are automatically determined during ECG analysis. Using the difference between the QRS and T axis, the frontal QRS-T angle was measured.

After examining the ECG leads, the P dispersion was computed minus the smallest P-wave duration from the highest P-wave duration [8]. Similarly, QT and QTc dispersion were calculated. Standard 24-h, three-channel (leads V1, V2, and V5) Holter ECG (Maynard, MA: Northeast Monitoring, Inc.) recordings were used. After manually adjusting R-R intervals, all recording was examined using a Century 2000/3000 HRV system (Maryland Heights, MO: Biomedical Systems). The patients had fasted overnight and stayed supine throughout the duration of the recordings. Intervals between normal and ectopic beats and between ectopic beats, as well as intervals assessed incorrectly due to artifacts, were eliminated from the analysis. The time-domain analysis examined the mean heart rate, the standard deviation of R-R intervals (SDNN), the proportion of NN50 divided by the total number of R-R intervals (pNN50), and the root mean square of successive differences (RMSSD). Applying the fast Fourier transform method, spectral measurements were obtained. Following the recommendations of the North American Society of Pacing and Electrophysiology, the recordings were obtained at an average of five separate five-minute intervals [9]. The total power was categorized as high frequency (HF) and low frequency (LF) components.

Statistical analysis

The SPSS program version 24.0 was used to conduct all of the analyses (Chicago, IL: IBM Corp.). Continuous variables were presented as mean±standard deviation or median (interquartile range). Categorical variables were presented as frequencies and percentages. Using the Student's t-test or the Mann-Whitney U test, continuous variables were compared. Nominal variables were compared utilizing the chi-squared test or Fisher's exact test. All tests were considered statistically significant if their p-value was less than 0.05.

## Results

The average age of the patient group was 38.9±5.8 years. The mean age of the control group was 37.4±5.3 years. Clinical parameters such as age, height, weight, and BMI did not differ significantly between the groups. The female gender was significantly higher in the study group than in the control group (p=0.032). The groups had no significant differences in heart rate, systolic blood pressure, or dyslipidemia. There were no appreciable differences between the groups for routine blood tests (Table 1).

**Table 1:** Clinical characteristics and laboratory parameters of patients. Data are presented as mean SD, number (percentage), or median (interquartile range). BMI: body mass index; SBP: systolic blood pressure; DL: dyslipidemia; AST: aspartate aminotransferase; ALT: alanine aminotransferase; LDL: low-density lipoprotein; HDL: high-density lipoprotein; CRP: c-reactive protein; eGFR: estimated glomerular filtration rate

Parameters	Study group, 90 (%)	Control group, 70 (%)	p-Value
Age (years)	38.9±5.8	37.4±5.3	0.331
Gender, female, n (%)	66 (73.3)	40 (57.1)	0.032
Weight (kg)	76.8±13.5	75.7±13.7	0.628
Height (cm)	166±7.1	165.5±7.5	0.789
BMI (kg/m²)	27.8±4.0	27.6±3.7	0.770
SBP (mmHg)	122.5±17.1	124.7±13.7	0.394
Heart rate (beat/minute)	89.6±18.4	90.3±18.6	0.800
DL, n (%)	27 (30)	29 (41.4)	0.133
White blood cell count (×10^3^µL)	9.6±3.2	9.9±3.6	0.596
Hemoglobin (g/dL)	13.3±1.9	13.9±2.2	0.091
AST (U/L)	18 (13)	20 (13.5)	0.321
ALT (U/L)	16 (17)	18 (17.2)	0.406
Glucose (mg/dL)	89 (30)	86.5 (29.5)	0.811
Total cholesterol (mg/dL)	186.9±55.9	178.3±44.1	0.289
Triglycerides (mg/dL)	156 (117.7)	142.5 (99.5)	0.135
LDL (mg/dL)	106.5±39.4	105.9±36.7	0.921
HDL (mg/dL)	39.9±8.8	41.3±13.0	0.426
CRP (mg/dL)	0.3 (0.3)	0.3 (0.2)	0.745
eGFR	95.4±30.8	99.8±22.3	0.314

P-wave maximum duration (Pmax), QT dispersion (QTd), and corrected QT dispersion (QTcd) were all high in the study (p<0.05). In terms of P-wave maximum duration (Pmin), P-wave dispersion (Pd), TpTe, corrected TpTe (TpTec), TpTe/QT, TpTe/QTc, and frontal QRS-T angle there were no significant differences across the two groups (Table 2).

**Table 2:** Comparison of ECG parameters between groups. Values are presented as mean±SD and median (interquartile range). Pmax: P-wave maximum duration; Pmin: P-wave minimum duration; Pd: P-wave dispersion; QTd: QT dispersion; QTcd: corrected QT dispersion; TpTe: T-peak to T-end; TpTec: corrected TpTe

ECG parameters	Study group, n (%)	Control group, n (%)	p-Value
Pmax (ms)	120.8±7.8	118.3±7.3	0.040
Pmin (ms)	72.7±8.2	72.0±11.0	0.646
Pd (ms)	51.1±9.1	48.3±13.6	0.129
QTd (ms)	54.0±6.6	50.3±8.1	0.002
QTcd (ms)	68.6±8.1	65.0±8.8	0.008
TpTe (ms)	75.6±18.1	75.8±14.4	0.678
TpTec (ms)	81.3±3.1	80.5±2.8	0.783
TpTe/QT	0.2±0.04	0.2±0.02	0.646
TpTe/QTc	0.19±0.05	0.19±0.33	0.813
Heart rate (beats/minute)	72±5	74±3	0.590
Frontal QRS-T angle	40±11	39±17	0.859

Comparing the results of 24-h Holter ECG recordings across the groups, no significant difference was seen (p=0.182). SDNN was substantially lower and LF and LF/HF were significantly higher in the study group (p<0.05). Between the groups, there was no obvious difference in pNN50 (p=0.49), RMSSD (p=0.55), or HF (p=0.44) (Table 3).

**Table 3:** Heart rate variability parameters of the groups. Data are expressed as mean±SD and median (interquartile range) as appropriate. SDNN: standard deviation of R-R intervals; RMSSD: root mean square of successive differences; pNN50: proportion of NN50 divided by the total number of R-R intervals; LF: low frequency; HF: high frequency

Parameters	Study group, (n=90)	Control group, (n=70)	p-Value
SDNN (ms)	102.6±43.2	140.3±18.9	<0.001
RMSSD (ms)	32.6±22.5	28.7±8.9	0.558
pNN50 (%)	10.6±9.2	8.0±5.3	0.492
LF (nu)	67.6±15.6	53.8±12.4	<0.001
HF (nu)	27.2±11.2	25.2±7.6	0.446
LF/HF	3.54±1.8	2.24±1.3	0.003

## Discussion

This study revealed that the QTd and QTc values of FM patients were higher than those of the control group. Although fibromyalgia patients complained of palpitations, Holter monitoring did not reveal any arrhythmias associated with clinical symptoms. Additionally, patients with FMS had significant autonomic nervous system dysfunction in favor of the sympathetic nervous system compared to the control group.

Autonomic nervous system disorders are characterized by sympathetic hyperactivity and parasympathetic dysfunction. Excessive stress, sleep disorders, sore points, and exercise intolerance are manifested [10,11]. Its pathogenesis is related to chronic stress, autonomic nervous system disorders, and neuroendocrine abnormalities [12]. According to Furlan et al., sympathetic hyperactivity exacerbates symptoms during repose [13]. Coronavirus disease 2019 (COVID-19) demonstrated that an increase in stress might exacerbate FMS symptoms, and FMS can develop following COVID-19 [14]. HRV is a useful indicator of the equilibrium between sympathetic and parasympathetic activity. HF, RMSSD, and pNN50 have been demonstrated to be markers of parasympathetic activity, while LF and SDNN are markers of sympathetic activity [15]. In RA patients, Anichkov et al. also reported decreased autonomic nervous function [16]. In our study, the time domain parameters pNN50 and RMSSD increased little; however, SDNN decreased significantly (p=0.49, p=0.55, and p<0.001, respectively). Nonetheless, we observed a considerable reduction in HF and a significant rise in LF and LF/HF ratio in patients with FMS, indicating that sympathetic modulation of the heart is predominant.

ECG parameters are used as non-invasive markers to estimate the risk of arrhythmia due to cardiac involvement in inflammatory diseases [17]. Pd has an independent predictive value for paroxysmal atrial fibrillation (PAF). Studies have shown that with prolonged QTd, ventricular arrhythmias are triggered, and sudden cardiovascular death develops [18]. In rheumatic disorders, such as rheumatoid arthritis, Behcet's disease, systemic sclerosis, and systemic lupus erythematosus, QTd was shown to be prolonged [19]. Electrolyte imbalance, heart failure, myocardial infarction, drug toxicity, anti-arrhythmic drugs, hypertrophic cardiomyopathy, and heart failure are other causes that prolonged QTd [20]. Therefore, these diseases were excluded from the study.

Several ventricular repolarization parameters, including TpTe time, TpTe/QT ratio, and QRS-T angle, were also examined. These values were discovered to be useful indicators of the expanded spread of ventricular repolarization. The TpTe time and TpTe/QT ratio were substantially longer and higher in the study group than in healthy controls, according to Zehir et al. [21]. Slightly more than 90 milliseconds (ms) of TpTec was associated with an almost threefold higher risk of sudden cardiac death [22]. TpTe, TpTe/QT ratio, and QRS-T angle were not significantly different across groups in our study. When compared to other electrocardiographic risk indicators, such as the QRS-T angle, it has been identified as a powerful and independent risk factor for cardiac morbidity and mortality [23]. Hnatkova et al. determined the QRS-T angle cutoff value to be 75 degrees, but Portland et al. calculated approximately 100 degrees [24,25]. In this study, the QRS-T angle cutoff value was not determined due to the lack of mortality or sudden cardiac death.

Holter monitoring is the best choice for assessing whether patients with prolonged ECG parameters may develop arrhythmia. Even though QT and QTc dispersions were considerable in our study group, dysrhythmia was not observed in the Holter recordings (p=0.182). Four patients in the research group demonstrated sinus tachycardia. One patient in each group was seen to have premature atrial contractions exceeding 10%. Our study has several limitations. The study population was relatively small. In the future, an implantable loop recorder could be used. Electrophysiological studies were not conducted.

## Conclusions

In our research, prolonged ECG parameters during Holter monitoring did not represent a significant risk for malignant arrhythmias. In addition, patients with FMS exhibited autonomic nervous system dysfunction in favor of sympathetic nervous system dysfunction. Therefore, it should be recognized that fibromyalgia does not raise the risk of malignant arrhythmias.

## References

[1] Wallace BI, Moore MN, Heisler AC (2022). Fibromyalgianess and glucocorticoid persistence among patients with rheumatoid arthritis. Rheumatology (Oxford).

[2] Covert L, Mater HV, Hechler BL (2021). Comprehensive management of rheumatic diseases affecting the temporomandibular joint. Diagnostics (Basel).

[3] Prados G, Miró E, Martínez MP, Sánchez AI, López S, Sáez G (2013). Fibromyalgia: gender differences and sleep-disordered breathing. Clin Exp Rheumatol.

[4] Heidari F, Afshari M, Moosazadeh M (2017). Prevalence of fibromyalgia in general population and patients, a systematic review and meta-analysis. Rheumatol Int.

[5] Mascarenhas RO, Souza MB, Oliveira MX, Lacerda AC, Mendonça VA, Henschke N, Oliveira VC (2021). Association of therapies with reduced pain and improved quality of life in patients with fibromyalgia: a systematic review and meta-analysis. JAMA Intern Med.

[6] Cabral CM, Miyamoto GC, Franco KF, Bosmans JE (2021). Economic evaluations of educational, physical, and psychological treatments for fibromyalgia: a systematic review with meta-analysis. Pain.

[7] D'Onghia M, Ciaffi J, Lisi L (2021). Fibromyalgia and obesity: a comprehensive systematic review and meta-analysis. Semin Arthritis Rheum.

[8] Corbo MD, Vitale E, Pesolo M (2022). Recent non-invasive parameters to identify subjects at high risk of sudden cardiac death. J Clin Med.

[9] Dilaveris PE, Gialafos EJ, Sideris SK (1998). Simple electrocardiographic markers for the prediction of paroxysmal idiopathic atrial fibrillation. Am Heart J.

[10] Aslan B, Ümit İ, Ferhat I (2021). Evaluation of the modified HASBLED score for prediction of in-hospital mortality in patients with COVID-19. Dicle Tıp Dergisi.

[11] Martínez-Lavín M (2021). Fibromyalgia in women: somatisation or stress-evoked, sex-dimorphic neuropathic pain?. Clin Exp Rheumatol.

[12] Kleykamp BA, Ferguson MC, McNicol E (2021). The prevalence of psychiatric and chronic pain comorbidities in fibromyalgia: an ACTTION systematic review. Semin Arthritis Rheum.

[13] Furlan R, Colombo S, Perego F (2005). Abnormalities of cardiovascular neural control and reduced orthostatic tolerance in patients with primary fibromyalgia. J Rheumatol.

[14] Mathkhor AJ, Abdullah AH, Khudhairy AS (2022). Post-COVID-19 fibromyalgia syndrome: a report of two cases. Ann Clin Anal Med.

[15] Emrah AC, Ozgul N, Donmez I, YALCİN OY, Alan S (2021). The pulmonary annular motion velocity assessed using tissue Doppler imaging could predict the proximal right coronary artery occlusion in patients with inferior myocardial infarction. Dicle Tıp Dergisi.

[16] Anichkov DA, Shostak NA, Ivanov DS (2007). Heart rate variability is related to disease activity and smoking in rheumatoid arthritis patients. Int J Clin Pract.

[17] Yilmaz R, Demirbag R (2005). P-wave dispersion in patients with stable coronary artery disease and its relationship with severity of the disease. J Electrocardiol.

[18] Lazzerini PE, Laghi-Pasini F, Capecchi PL, Boutjdir M (2022). Emerging risk factors for QT interval prolongation and torsades de pointes. Torsades de Pointes.

[19] Zhang S, Hu J, An S (2021). Prevalence and relevant factors of positive RF in brucellosis patients with arthralgia. PLoS Negl Trop Dis.

[20] Sanad AM, Ghazal KH, Eltahlawi MA, Abdelwahed AT (2021). An overview of QT dispersion finding in cardiac patients, review article. Egypt J Hosp Med.

[21] Zehir R, Karabay CY, Kalaycı A, Akgün T, Kılıçgedik A, Kırma C (2015). Evaluation of Tpe interval and Tpe/QT ratio in patients with slow coronary flow. Anatol J Cardiol.

[22] Chua KC, Rusinaru C, Reinier K (2016). Tpeak-to-tend interval corrected for heart rate: a more precise measure of increased sudden death risk?. Heart Rhythm.

[23] Poulikakos D, Hnatkova K, Banerjee D, Malik M (2018). Association of QRS-T angle and heart rate variability with major cardiac events and mortality in hemodialysis patients. Ann Noninvasive Electrocardiol.

[24] Hnatkova K, Seegers J, Barthel P (2018). Clinical value of different QRS-T angle expressions. Europace.

[25] Jaber S, Nussinovitch U, Stahi T, Arnson Y (2022). Association between T wave morphology parameters and abnormal cardiac SPECT imaging. J Electrocardiol.

